# Understanding the landscape of nursing data

**DOI:** 10.1097/nmg.0000000000000376

**Published:** 2026-05-29

**Authors:** Angela Pascale

**Affiliations:** **Angela Pascale** is a Research Analyst at Press Ganey Associates LLC in South Bend, IN.

## Abstract

This article is the second in a four-part Data Toolkit series designed to help nurse managers better understand and use nursing data. The first article (“How data visualization elevates nursing performance insight,” March/April 2026, pp. 9-12) covered basic data concepts and how to read metrics. This article builds on that foundation by explaining the main types of nursing data and how they connect to each other to help make sense of performance.

**Figure:**
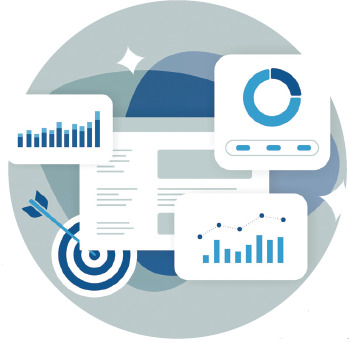
No caption available.

**THIS COLUMN INTRODUCES** the core domains of nursing data and examines how interpreting patterns across these domains supports a more complete understanding of performance. Nurse managers (NMs) routinely work with a wide range of nursing data, including patient outcomes, workforce metrics, and structural measures that collectively reflect the results of care, how care is delivered, and the conditions under which care occurs. Each type of nursing data—patient outcomes, workforce metrics, and structural measures—offers a different lens on unit performance, and data's value is strongest when both the meaning of individual measures and their connections are considered together. Viewing measures across nursing data domains in an integrated way supports clearer interpretation and more focused strategic action. It also sets the foundation for effective visualization, helping to ensure that visual displays highlight the relationships that matter and communicate nursing's impact with clarity.

This column focuses on building a clear understanding of the core nursing data domains—what each one shows on its own and how combining them leads to useful insights that strengthen how visualizations communicate nursing impact. To help interpret patterns identified across different types of data, the next section describes the main nursing data domains that influence performance.

## CORE DOMAINS OF NURSING DATA

**FIGURE 1.**
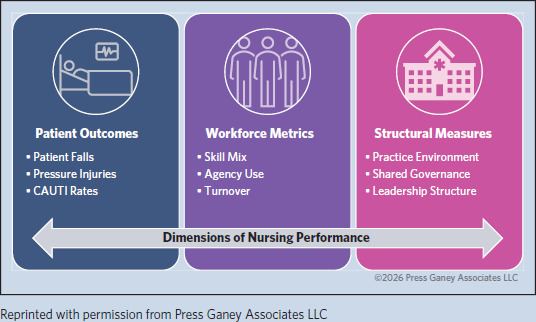
Core nursing data domains

Nursing data are organized across domains that reflect different aspects of care delivery and performance. Although the specific measures available to NMs differ across organizations, nursing data are often structured within three core domains: patient outcomes, workforce metrics, and structural measures (see Figure 1).

### Patient outcomes

Patient outcomes refer to outcomes that are influenced by nursing care. These measures reflect the results of care processes in areas where nursing assessment, surveillance, coordination, and intervention play a substantial role. Common examples include patient falls, pressure injuries, and hospital-acquired infections.

Outcome measures capture what occurred during patient care and represent the observable results of clinical practice. These measures function as primary indicators of quality and safety performance by quantifying patient-level events and outcomes. In performance monitoring, they're used to evaluate trends, benchmark against peers, and assess quality improvement efforts.

### Workforce metrics

Workforce metrics describe the characteristics of the nursing workforce and how nursing care is staffed. These measures capture elements such as staffing levels, registered nurse (RN) hours per patient day, skill mix, agency utilization, and turnover.

Workforce data reflect the nursing personnel available to deliver care and the stability of the workforce over time. These measures are used to examine staffing capacity, variation in staffing patterns, and workforce sustainability. Unlike outcome measures, workforce metrics don't describe patient results; rather, they reflect how nursing care is resourced and distributed.

### Structural measures

Structural measures reflect the resources and systems in place to support care before it happens. Structure encompasses the work environment, organizational systems, leadership structures, governance models, and resources that support and shape care delivery. In practice, structural measures may include whether a unit has an active shared governance council, or practice environment survey scores, with data typically captured through administrative records or standardized staff surveys.

Structural measures describe the infrastructure surrounding nursing practice. They don't capture patient outcomes directly, nor do they quantify staffing levels, but instead reflect the broader conditions that influence how care is organized and sustained.

## WHY METRICS IN ISOLATION CAN BE MISLEADING

Although nursing data are organized into distinct domains, each domain reflects only one dimension of performance. Patient outcomes describe what occurred during care, workforce metrics characterize how care is staffed, and structural measures reflect the organizational conditions that shape practice. When interpreted independently, however, each provides only a partial view.

For example, an increase in patient falls may signal a quality concern, but outcome data alone don't reveal whether contributing factors include staffing variability, skill mix changes, or broader environment conditions. Similarly, shifts in turnover or agency utilization describe workforce instability but don't, in isolation, demonstrate how those changes are influencing patient outcomes. Structural measures may indicate strong governance or a supportive work environment; yet without accompanying outcome and workforce data, their practical implications remain unclear.

Evaluating a single metric without considering related domains can therefore result in an incomplete understanding of performance dynamics. Nursing performance is inherently multidimensional, and meaningful interpretation requires understanding how different types of data inform and contextualize one another.

## MAKING CONNECTIONS ACROSS NURSING DATA

Examining nursing data across domains allows NMs to move beyond isolated interpretation and toward a more integrated understanding of performance and its underlying drivers. Making connections across different nursing data domains involves intentionally exploring how outcome, workforce, and structural measures relate to one another.

Cross-domain interpretation reveals patterns in how performance signals align or diverge. Table 1 provides examples of these cross-domain patterns and describes how they may appear in practice. These cross-domain patterns don't establish causation, nor do they provide definitive explanations. Rather, they offer a structured approach for interpreting how cross-domain signals contribute to a more complete understanding of performance and inform strategic action.

**TABLE 1:** Cross-domain performance pattern examples

Cross-domain pattern	What this pattern may indicate	Illustrative practice example
Coordinated change	Outcome and workforce or structural measures shift during the same reporting period. This shift reflects concurrent change (regardless of direction).	RNHPPD decreased in the same quarter that fall rates increased.
Divergent pattern	Workforce or structural measures change, but outcomes don't shift in the same reporting period. No immediate outcome response is evident.	RNHPPD increased but fall rates remained stable during the same reporting period.
Lagged relationship	Outcome changes occur in a subsequent period following workforce or structural shifts. This suggests a delayed association.	RNHPPD decreased in Quarter 1, and fall rates increased in Quarter 2.
No observable alignment	Measures vary across reporting periods without consistent timing or directional alignment. No clear cross-domain pattern is evident.	RNHPPD levels and fall rates both changed over time, but increases and decreases didn't consistently occur together or in sequence.

**Key:** RNHPPD, registered nurse hours per patient day

### Using cross-domain views to clarify performance patterns

In practice, cross-domain interpretation helps distinguish whether observed changes reflect isolated variation or broader performance dynamics. Viewing metrics in relation to one another provides clearer insight into underlying conditions influencing care delivery. By clarifying how performance signals align, diverge, or sequence across domains, NMs can more confidently determine where focused operational review is warranted.

To illustrate how interpretation shifts when metrics are viewed relationally rather than in isolation, Table 2 presents examples. These comparisons demonstrate how examining patterns across outcome, workforce, and structural domains can change how observed trends are understood and the strategic conclusions drawn.

**TABLE 2:** How cross-domain linkage changes interpretation of isolated metrics

Looking at a single metric in isolation	What cross-domain linkage reveals
Fall rate increased	RNHPPD decreased during the same period, indicating concurrent staffing reduction may be influencing outcome variation.
Turnover high	Practice environment scores declined during the same period, indicating potential strain in the work environment.
Skill mix changed	A subsequent increase in fall rates occurred in the following quarter, indicating a potential delayed effect of staffing changes on fall rates.

**Key:** RNHPPD, registered nurse hours per patient day

Understanding the landscape of nursing data and establishing relational context across domains creates a stronger foundation for interpretation and communication. This foundation strengthens the story an NM can tell when explaining what is changing, why it matters, and what should happen next, while clearly reflecting nursing's impact. NMs can then translate routine metrics into prioritized action, distinguishing between issues that require staffing adjustment, organizational intervention, or closer outcome monitoring.

## TRANSLATING DOMAIN-LEVEL DATA INTO CLEAR VISUALS

Understanding nursing data conceptually is essential, but insights on cross-domains patterns and relationships must be translated into clear visualizations that guide interpretation and decision-making. The *Supplemental Excel Toolkit* (http://links.lww.com/NMT/A12) supports this next step by providing structured visuals for each core nursing data domain, including outcomes, workforce, and structural measures, alongside cross-domain linkage views.

Each tab allows NMs to enter local data and observe how patterns appear within that domain's visual structure. Working directly with these examples strengthens fluency in how different types of nursing data are represented visually and reinforces how visual framing shapes interpretation.

The toolkit is intended for NMs as a repeatable reference to support interpretation of nursing performance data over time. It's designed to help leaders recognize patterns; spot priorities; and connect outcome, workforce, and structural data. In doing so, the toolkit strengthens NMs' ability to communicate key points and helps frame insights for business case development, resource needs, or performance improvement discussions.

## CONNECTING DATA DOMAINS

For NMs, strong performance interpretation is strengthened when different types of nursing data are viewed in relation to one another. This column outlined the core data domains of patient outcomes, workforce metrics, and structural measures, and explained how examining them together provides deeper context than any single metric alone. By approaching data with a cross-domain perspective, NMs can better recognize meaningful patterns, facilitate data-informed discussions, and prioritize actions aligned with unit needs.

